# Data on phylogenetic analyses of gazelles (genus *Gazella*) based on mitochondrial and nuclear intron markers

**DOI:** 10.1016/j.dib.2016.02.062

**Published:** 2016-03-02

**Authors:** Hannes Lerp, Sebastian Klaus, Stefanie Allgöwer, Torsten Wronski, Markus Pfenninger, Martin Plath

**Affiliations:** a Natural History Collections, Museum Wiesbaden, Friedrich-Ebert-Allee 2, 65185 Wiesbaden, Germany; b Department of Ecology and Evolution, J.W. Goethe-University Frankfurt, Max-von-Laue-Str. 13, 60438 Frankfurt am Main, Germany; c Zoological Society of London, Conservation Programs, Regent’s Park, London NW1 4RY, United Kingdom; d King Khalid Wildlife Research Center, Saudi Wildlife Authority, P.O. Box 61681, Riyadh 11575, Kingdom of Saudi Arabia; e Molecular Ecology Group, Senckenberg Biodiversity and Climate Research Centre (BiK-F), 60325 Frankfurt am Main, Germany; f College of Animal Science and Technology, Northwest A&F University, Yangling 712100, PR China

## Abstract

The data provided is related to the article “Phylogenetic analyses of gazelles reveal repeated transitions of key ecological traits and provide novel insights into the origin of the genus *Gazella*” [1]. The data is based on 48 tissue samples of all nine extant species of the genus *Gazella*, namely *Gazella gazella*, *Gazella arabica*, *Gazella bennettii*, *Gazella cuvieri*, *Gazella dorcas*, *Gazella leptoceros*, *Gazella marica*, *Gazella spekei*, and *Gazella subgutturosa* and four related taxa (*Saiga tatarica*, *Antidorcas marsupialis*, *Antilope cervicapra* and *Eudorcas rufifrons*). It comprises alignments of sequences of a cytochrome *b* data set and of six nuclear intron markers. For the latter new primers were designed based on cattle and sheep genomes. Based on these alignments phylogenetic trees were inferred using Bayesian Inference and Maximum Likelihood methods. Furthermore, ancestral character states (inferred with BayesTraits 1.0) and ancestral ranges based on a Dispersal-Extinction-Cladogenesis model were estimated and results׳ files were stored within this article.

## 1 Specifications table

**Table:** 

Subject area	Biology, genetics and genomics
More specific subject area	Phylogenetics and phylogenomics
Type of data	Tables, primer sequences, sequence alignments, phylogenetic trees, ancestral character state estimation and ancestral ranges estimation.
How data was acquired	Primers were designed using the Oligonucleotide Properties Calculator [2]. Sequences were aligned with MUSCLE [3].
Phylogenetic trees were inferred with BEAST MC3 1.7.5 [4] and RAxML 8.0.14 [5].
Ancestral character state estimation was conducted with BayesTraits multistate 1.0 [6].
Ancestral ranges were estimated based on a Dispersal-Extinction-Cladogenesis (DEC)-model implemented in Lagrange v. 20130526 [7].
Data format	Analyzed
Experimental factors	Sample types used for DNA extraction were tissue, skin, blood and hairs and were extracted using Qiagen DNeasy blood and tissue kit according to the manufacturer’s protocol.
Experimental features	We sampled gazelle species from a wide geographic range to cover as much of the extant diversity as possible.
Data source location	Samples were collected in Israel, Saudi Arabia, Oman, Chad, Algeria, Sudan, Tunisia, Mongolia, Pakistan, and from captive breeding stocks
Data accessibility	Data is available within the article.

## 2 Value of the data

• New nuclear intron primers for phylogenetic investigations of closely related bovid species.
• Data provide phylogenetic insight into the genus *Gazella.*
• Ancestral character state and ancestral range information for the genus *Gazella* were inferred with this data.

## 3 Data

Data provided with this article are newly established primer sequences of nuclear intron markers for bovids and sequence alignments of the respective markers and Cyt *b* including species from the genera *Gazella*, *Eudorcas*, *Antilope*, *Saiga* and *Antidorcas*. Furthermore, phylogenetic tree files and result files from analyses of ancestral character state estimation and ancestral ranges estimation for the genus *Gazella* are shared.

## 4 Experimental design, materials and methods

### 4.1 PCR primer design

We designed new nuclear primers for the amplification of introns of the nuclear encoded genes *zinc finger protein 618* (ZNF618), *epidermal growth factor receptor substrate 15-like 1* (EPS15L1), *SPARC-related modular calcium-binding protein 1* (SMOC1), *pantothenate kinase 4* (PANK4), NACHT, LRR and *PYD domains-containing protein 2* (NLRP2) and *chromodomain-helicase-DNA-binding protein 2* (CHD2; Table 1). We used the sheep (*Ovis aries*) genome, available on the website of the international sheep genomics consortium (http://www.livestockgenomics.csiro.au/sheep/oar1.0.php), and cattle (*Bos taurus*) genome, available from the Ensembl genome database (http://www.ensembl.org/Bos_taurus/Info/Index). We searched the sheep genome for annotated protein-coding genes and used the provided Swiss-Prot number to search for the corresponding gene sequences in the cattle genome. If those sequences contained introns of a length between 400 and 1000 bp, we assembled the exons of the respective gene with the complete gene sequence of sheep using Geneious Pro 5.4.2 (Biomatters Ltd., available from http://www.geneious.com). Primers were subsequently designed according to conserved regions of the exons of cattle and sheep in a way that the resulting sequences stretched across at least one intron. To avoid linkage disequilibrium we only used genes on different chromosomes. Primers were designed using the Oligonucleotide Properties Calculator [2] and the reverse complement converter (http://www.bioinformatics.org/sms/rev_comp.html). All primers were synthesized by Eurofins MWG Synthesis GmbH.

### 4.2 Sequence alignments

DNA was extracted using the Qiagen DNeasy blood and tissue kit according to the manufacturer’s protocol. Sequences were obtained by Sanger sequencing, and newly established sequences were deposited in GenBank (Table 2). We aligned sequences with MUSCLE ([3]; gapopen=−400; gapextend=−200). In total, the concatenated alignment consisted of 4,623 nucleotides. The Cyt *b* gene partition was translated into amino acid sequences and checked for stop codons that would indicate potential pseudogenes. The alignments for the six nuclear introns of the genes *ZNF618*, *EPS15L1, SMOC1*, *PANK4*, *NLRP2*, *CHD2* and the mitochondrial Cytochrome *b* gene are supplemented to this article (Lerp_et_al_Gazella_{gene code}_alignment.nexus).

### 4.3 Phylogenetic analyses

Phylogeny and divergence times were estimated with a Bayesian approach in BEAST MC3 1.7.5 [4]. Additionally, we inferred a species tree using a coalescence approach on the multiple loci as implemented in the ^*^BEAST algorithm [8] that we used for subsequent ancestral character (1000 trees) and range (maximum clade credibility tree) estimation. Molecular clock rates and substitution schemes were unlinked between partitions. We inferred the most likely substitution model for each marker using jModelTest 2.1.3 [9], considering models with equal/unequal base frequencies and with/without rate variation among sites (base tree for likelihood calculations=ML tree; tree topology search operation=NNI; the best model was inferred based on the Akaike Information Criterion). This resulted in a HKY+G model of sequence evolution for all genes except for *PANK4* with a HKY model. We applied a Yule tree prior to account for independently evolving lineages. We chose an uncorrelated log-normal relaxed molecular clock using an external substitution rate for the Cyt*b* gene (normally distributed rate with a mean of 1.50±0.15% per Ma; 5–95% interquantile range: 1.25–1.75% per Ma; [10]). This rate was estimated based on four different alignments of primate protein-coding mitochondrial sequences and fossil calibration points for six primate data sets using a Bayesian approach [10]. For the more conserved nuclear genes reliable external rates were not available, and so we assumed a very broad exponentially distributed prior with a mean of 0.01% per Ma (5–95% interquantile range: 0.01–0.30% per Ma).

We ran three chains for 50 M iterations, sampling every 10,000th iteration. Convergence of sampled parameters and potential autocorrelations (effective sampling size for all parameters>200) were investigated in Tracer 1.6 [11]. We discarded the first 10% of sampled trees as burn-in. The maximum clade credibility tree was chosen and parameter values annotated using TreeAnnotator (part of the BEAST package). The resulting substitution rates were 0.97% per Ma for Cyt *b* (95% credibility interval, CI: 0.05–1.45%), 0.12% per Ma for *EPS15L1* (CI: 0.05–0.19%), 0.17% per Ma for *NLRP2* (CI: 0.08–0.27%), 0.16% per Ma for *SMOC1* (CI: 0.04–0.32%), 0.21% per Ma for *ZNF618* (CI: 0.1–0.32%) and 0.11% per Ma for *PANK4* (CI: 0.05–0.18%).

To confirm the tree topology calculated in BEAST we also analyzed the concatenated data set with a Maximum Likelihood (ML) approach. ML-analysis was performed with RAxML 8.0.14 [5] under a GTR+Γ model that was unlinked for all partitions. Support of nodes was assessed with 1,000 bootstrap replicates. Phylogenetic (Bayesian and ML) and species trees are Supplemented to this article (Lerp_et_al_Gazella_phylogeny_{program}.nwk and Lerp_et_al_Gazella_Species_Tree_starBEAST.nwk).

### 4.4 Ancestral character state estimation

We estimated ancestral characters for ecological and behavioral traits using a Bayesian approach to character evolution in BayesTraits multistate 1.0 [6]. The analysis was conducted with 1000 randomly selected post-burn-in trees to account for uncertainty in phylogenetic reconstruction; outgroups were removed with exception of *Antilope cervicapra* (the sister group to *Gazella*, see [1]). We estimated ancestral character states for three key ecological/behavioral traits: habitat type (mountainous vs. plain-dwelling), group size (small groups<15 individuals vs. large herds), and movement patterns (sedentary vs. migratory; see input files). In addition, we reconstructed ancestral character states for presence or absence of horns in females, and the occurrence of twinning (see Table S2 in [1]). We ran the analysis for 20 M iterations, sampling every 10,000th iteration and discarding the first 10% as burn-in. To specify the range of values used to seed the prior distribution, we applied an exponential hyperprior with a mean ranging from 0.0 to 0.5 and a rate deviation of seven (twinning=2, female horns=6), resulting in mean acceptance rates between 20% and 40%. To further corroborate the ancestral state in the most recent common ancestor (MRCA) of the genus *Gazella* we additionally applied a model testing approach. In separate runs – with the general MCMC setting as described above – we constrained the ancestral condition of the MRCA of *Gazella* to each of the alternative states and compared the harmonic mean of likelihoods (as an estimator of marginal likelihoods) using the Bayes factor (BF). As harmonic means tend to be unstable, we repeated each run five times and calculated the BF from the arithmetic means. Result files of the ancestral character state estimation (ACSE) are supplemented to this article (Lerp_et_al_Gazella_ACSE_{trait}.txt).

### 4.5 Biogeography

To estimate ancestral ranges based on a Dispersal-Extinction-Cladogenesis (DEC) model as implemented in the software Lagrange v. 20130526 [7] the species tree (maximum clade credibility tree with median heights) obtained through Bayesian inference was used as phylogenetic input. Species were assigned to one of four discrete geographic areas: (a) Africa, (b) Middle East, (c) Central Asia, and (d) India (Figure 3 in [1]). We did not take into account the distribution data of the more distant outgroups, but included the genus *Antilope* as the nearest extant relative of the genus *Gazella*.

To test for the direction of dispersal we calculated three models of range evolution: without constrained dispersal (*H*_0_); with dispersal only from Africa to Asia (i.e., Middle East, Central Asia, India) allowed (Afr→As), and a third model allowing only dispersal from Asia to Africa (As→Afr). We compared the resulting global maximum likelihood at the root nodes and the AIC between models (Table 1 in [1]). In all three models, Africa was assumed adjacent only to the Middle East, while adjacency between the three Asian ranges was not constrained. Model results can be found within this article (Lerp_et_al_Gazella_DEC_H0.txt, Lerp_et_al_Gazella_DEC_Afr→As.txt, Lerp_et_al_Gazella_DEC_As→Afr.txt).

## Figures and Tables

**Table 1:** Newly designed intron primers for bovid species with chromosome number of sheep and cattle, Swiss-Prot number, melting (*T*_M_) and annealing temperatures, amplification lengths and GC contents.

Primer name	Protein	Chromosome number sheep	Chromosome number cattle	Swiss-Prot number	Primer forward	Reverse
ZNF618	Zinc finger protein 618	Chr 2	Chr 8	Q5T7W0	TCC TAT GAG TGT GGA ATC TGT GG	TCT CCT GAG GTG GCT TCA GTG
EPS15L1	Epidermal growth factor receptor substrate 15-like 1	Chr 5	Chr 7	A7MB30	CAA AGA CCA GTT CGC GTT AGC TA	TCC CCC GAT CCA AGA GTG CT
Smoc1	SPARC-related modular calcium-binding protein 1	Chr 7	Chr 10	Q9H4F8	TGG CTA CTG CTG GTG TGT GC	CCTGTCCTTGAAGGGGTCCT
PANK4	Pantothenate kinase 4	Chr 12	Chr 16	Q4R4U1	ACT GGG GGT GGG GCA TAC AA	GGT CAT CAC ATC CTC CTT GTC AA
NLRP2	NACHT, LRR and PYD domains-containing protein 2	Chr 14	Chr 18	Q9NX02	CAG TCC CTC ACA TGC TTG AAC	CAG TTT CAC CCC ACG ATC TC

Primer name	*T*_M_ (salt adjusted) [°C]		Annealing-temp. [°C] ( *T*_M_ − 5 °C)	Amplification length [bp]	GC-content [%]	
	Forward	Reverse			Forward	Reverse
ZNF618	60.6	61.8	61→56	679	48	57
EPS15L1	60.6	61.4	61→56	365	48	60
Smoc1	61.4	61.4	61→56	659	60	60
PANK4	61.4	60.6	61→56	443	60	48
NLRP2	59.8	59.4	59→54	532	52	55
CHD2	61.0	61.8	61→56	733	46	57

**Table 2:** Accession numbers of sequences used in this study.

**Sample ID**	**CHD2**	**EPS15L1**	**NLRP2**	**PANK4**	**SMOC1**	**ZNF618**	**Cyt*****b***
GH1	KU560704	KU560659	KU560746	KU560790	KU560880	KU560837	KU560629
TAUM 11861	KU560705	KU560660	KU560747	KU560791	KU560881	KU560838	KC188775
TAUM 12479	KU560706	KU560661	KU560748	KU560792	KU560882	KU560839	KC188774
TAUM 10170	KU560707	KU560662	KU560749	KU560793	KU560883	KU560840	KC188740
TAUM 11048	KU560708	KU560663	KU560750	KU560794	KU560884	KU560841	KC188759
GGF41	KU560709	KU560664	KU560751	KU560795	KU560885	KU560842	KU560630
OmanI	KU560710	KU560665	KU560752	KU560796	KU560886	KU560843	KU560648
3455	KU560711	KU560666	KU560753	KU560797	–	KU560844	KU560649
182	–	–	–	KU560798	KU560887	–	JN410348
3463	KU560712	KU560667	KU560754	KU560799	KU560888	–	KU560631
3466	KU560713	KU560668	KU560755	KU560800	–	KU560845	KU560650
3467	KU560714	KU560669	KU560756	KU560801	–	KU560846	KU560632
3469	KU560715	KU560670	KU560757	KU560802	–	KU560847	KU560651
Chad19	KU560716	KU560671	KU560758	KU560803	KU560889	–	JN410237
Chad7	KU560717	KU560672	–	KU560804	KU560890	–	JN410235
2866	KU560718	KU560673	KU560759	KU560805	KU560891	KU560848	JN410252
3564	KU560719	KU560674	KU560760	KU560806	KU560892	KU560849	JN410230
AWWP 9159	KU560720	KU560675	KU560761	KU560807	KU560893	KU560850	JN410319
PCGD59	KU560721	KU560676	KU560762	KU560808	KU560894	KU560851	JN410251
PCGD1	–	KU560677	–	KU560809	KU560895	KU560852	JN410257
3261	KU560722	KU560678	KU560763	KU560810	KU560896	KU560853	JN410255
Mongo	KU560723	KU560679	KU560764	KU560811	KU560897	–	KU560652
AWWP 9053	KU560724	KU560680	KU560765	KU560812	KU560898	KU560854	KU560653
583	KU560725	KU560681	KU560766	KU560813	KU560899	KU560855	KU560633
7	KU560726	KU560682	KU560767	KU560814	KU560900	KU560856	JN410357
9	KU560727	KU560683	KU560768	KU560815	KU560901	KU560857	JN410341
6	KU560728	KU560684	KU560769	KU560816	KU560902	KU560858	JN410340
10	–	–	KU560770	KU560817	KU560903	KU560859	KU560634
2887	KU560729	KU560685	KU560771	KU560818	KU560904	KU560860	KU560635
2885	KU560730	KU560686	KU560772	KU560819	KU560905	KU560861	KU560636
781	KU560731	KU560687	KU560773	KU560820	KU560906	KU560862	JN410345
782	KU560732	KU560688	KU560774	KU560821	KU560907	KU560863	JN410344
75	KU560733	–	KU560775	KU560822	KU560908	KU560864	KU560654
90	KU560734	KU560689	–	KU560823	KU560909	KU560865	KU560655
271	–	KU560690	KU560776	KU560824	KU560910	KU560866	KU560656
AWWP 7895	–	KU560691	KU560777	KU560825	–	KU560867	KU560657
AWWP 9055	KU560735	KU560692	KU560778	KU560826	–	KU560868	KU560637
AWWP 8397	–	KU560693	KU560779	KU560827	–	KU560869	KU560638
AWWP 7238	KU560736	KU560694	KU560780	–	–	KU560870	KU560658
OZ1	KU560737	KU560695	KU560781	KU560828	KU560911	KU560871	KU560639
OZ2	KU560738	KU560696	KU560782	KU560829	KU560912	KU560872	KU560640
OZ3	KU560739	KU560697	KU560783	KU560830	KU560913	KU560873	KU560641
OZ4	KU560740	KU560698	KU560784	KU560831	–	KU560874	KU560642
S06	KU560741	KU560699	KU560785	KU560832	–	KU560875	KU560643
S08	KU560742	KU560700	KU560786	KU560833	–	KU560876	KU560644
S10	KU560743	KU560701	KU560787	KU560834	–	KU560877	KU560645
S12	KU560744	KU560702	KU560788	KU560835		KU560878	KU560646
SB	KU560745	KU560703	KU560789	KU560836	–	KU560879	KU560647

## Footnotes

Appendix A Supplementary data associated with this article can be found in the online version at 10.1016/j.dib.2016.02.062.
